# The Effect of Restorative Material Type, Thickness, Curing Mode, and Post-Irradiation Time on the Degree of Conversion of a Dual-Cure Resin Cement †

**DOI:** 10.3390/polym18172088

**Published:** 2026-08-28

**Authors:** Sinem Kantarcioglu, Merve Sena Ekinci, Neva Secilmis

**Affiliations:** 1 Department of Prosthodontics, Faculty of Dentistry, Gaziantep University, 27410 Gaziantep, Türkiye; sinem.kantarcioglu1@gmail.com; 2 Department of Prosthodontics, Adiyaman University, 02040 Adiyaman, Türkiye; 3 Independent Researcher, Gaziantep, Türkiye; nevasecilmis0@gmail.com

**Keywords:** degree of conversion, FT-IR/ATR, CAD/CAM, 3D printing, dual-cure resin cement

## Abstract

This in vitro study evaluated the effects of restorative material type, thickness, curing mode, and post-irradiation time on the degree of conversion (DC) of a dual-cure resin cement. Specimens of VITA Suprinity (VS), VITA Enamic (VE), Lava Ultimate (LU), and Saremco Print Crowntec (SC) were prepared at thicknesses of 1.0 and 1.5 mm. A dual-cure resin cement was placed beneath each specimen and light-cured through the specimen using a VALO X curing light in Standard Power (SP) or Xtra Power (XP) mode (*n* = 8). DC was measured by FT-IR/ATR spectroscopy at days 1 and 10. The normality and homogeneity of variances were assessed using the Shapiro–Wilk and Levene tests, and data were analyzed using mixed-design repeated-measures ANOVA and Tukey HSD tests (α = 0.05). Material type and thickness significantly affected DC (*p* < 0.001), whereas curing mode did not (*p* = 0.110). Post-irradiation time significantly affected DC (*p* < 0.001), and a significant material–thickness interaction was found (*p* = 0.043). Within the limitations of this study, DC was affected by material type, thickness, and post-irradiation time, but not by curing mode.

## 1. Introduction

Computer-aided design and computer-aided manufacturing (CAD/CAM) systems have become widely used in the fabrication of indirect restorations [1]. These restorations can be fabricated using subtractive manufacturing, which involves milling prefabricated blocks, or additive manufacturing, which involves the layer-by-layer fabrication of three-dimensional (3D) objects [2]. These manufacturing technologies have expanded the range of restorative materials available for CAD/CAM applications. These materials include feldspathic ceramics, lithium disilicate ceramics, zirconia-reinforced lithium silicate ceramics, resin nanoceramics, glass ceramics in a resin-interpenetrating matrix, zirconia-silica ceramics in a resin-interpenetrating matrix, and resin-based materials manufactured using 3D printing [1,2]. These materials differ in their composition, optical and mechanical properties, and clinical performance; therefore, appropriate material selection is essential for the long-term success of indirect restorations [3].

The long-term clinical performance of indirect restorations depends on appropriate restorative material selection as well as the performance of the resin cement. One of the principal factors determining resin cement performance is the degree of polymerization achieved. Inadequate polymerization may increase water sorption and solubility, reduce bond strength, and compromise marginal integrity, thereby increasing the risk of adhesive failure, secondary caries, and restoration fracture [4,5]. Furthermore, unreacted monomers released from inadequately polymerized resin cements may exert cytotoxic effects on oral tissues, induce oxidative stress, and alter the expression of inflammatory mediators [6,7,8].

Resin cements are available in light-cured, self-cured, and dual-cured formulations according to their polymerization mechanisms [3,9,10]. Because the polymerization of light-cured resin cements depends entirely on light activation, attenuation of the curing light by the overlying restorative material may adversely affect their degree of conversion (DC) [11]. For this reason, achieving adequate polymerization can be challenging in indirect restorations where light transmission is limited [3,12]. Dual-cure resin cements, which utilize both light activation and chemical polymerization mechanisms, can achieve higher DC in areas where light cannot penetrate sufficiently and offer more predictable polymerization [13,14]. Because post-cure polymerization of dual-cure resin cements may continue for several days or weeks depending on the material, their DC may vary according to the time elapsed after light activation. Consequently, evaluating DC at different post-irradiation time points may provide a more comprehensive assessment of the polymerization behavior of these materials than a single-time-point measurement [5,12].

As the curing light passes through the restoration, part of the incident light is absorbed or scattered, whereas the remaining portion reaches the underlying resin cement. The optical power transmitted through the restoration per unit area is termed transmitted irradiance, is expressed in mW/cm^2^, and may influence photoinitiator activation and DC [15]. Restorative material characteristics, including thickness, color, chemical composition, and translucency, can affect the amount of light reaching the underlying resin cement and, consequently, its DC [16]. Translucency reflects the extent of light absorption and scattering within a material and is directly influenced by material thickness [17,18]. The translucency of restorative materials is commonly evaluated using the translucency parameter (TP), defined as the color difference obtained from measurements over black and white backgrounds [19]. When alternative background conditions are used, the CIEDE2000-based relative translucency parameter (RTP_00_) provides a perceptually relevant assessment of translucency differences and masking ability [20].

Light-curing-unit-related parameters can also influence DC. The wavelength and irradiance of the curing unit, the curing mode, irradiation time, and application distance collectively determine the amount of energy delivered to the resin cement, thereby influencing its DC [21,22,23,24]. Radiant exposure (J/cm^2^), defined as the time integral of irradiance, quantifies the total radiant energy delivered per unit area. It therefore provides a more comprehensive characterization of light-curing protocols, particularly when comparing curing modes that differ in irradiance, exposure duration, and output profile [21]. Among these parameters, curing mode is of particular relevance for multi-wavelength LED curing units such as the VALO X (Ultradent Products, South Jordan, UT, USA). The VALO X provides two distinct power modes: Standard Power (SP), which delivers a continuous curing cycle, and Xtra Power (XP), which delivers a shorter, pulsed curing cycle with brief automatic rest intervals between pulses. Although XP mode substantially reduces curing time, its effect on the polymerization efficiency of resin cements beneath restorative materials of different thicknesses and translucencies has yet to be clearly established [25].

Numerous studies have evaluated the effects of the type and thickness of CAD/CAM restorative materials on the DC of resin cements and compared different light-exposure durations [3,5,12,13,14,16,22,23,24,26,27,28,29,30,31,32,33]. However, studies examining the effect of the light transmission properties of 3D-printed restorative materials on the DC of dual-cure resin cements are limited [34]. The effects of curing protocols combining different irradiance levels and exposure durations, particularly SP and XP modes, on the DC of resin cements have not been sufficiently investigated. Moreover, the combined effects of restorative material type, material thickness, curing protocol, and post-irradiation time on the DC of dual-cure resin cements remain unclear.

The aim of this study was to evaluate the effects of restorative material type, material thickness, curing mode (SP and XP), and post-irradiation time on the DC of a dual-cure resin cement beneath restorative materials fabricated using milling and 3D printing technologies. The null hypothesis was that restorative material type, material thickness, curing mode, and post-irradiation time would have no significant effect on the DC of the dual-cure resin cement.

## 2. Materials and Methods

An a priori power analysis was performed using G*Power 3.1.9.7 (Heinrich Heine University, Düsseldorf, Germany) based on a conservative effect size of f = 0.40 derived from a previous study comparing DC through 1.0- and 1.5-mm-thick restorative materials [29]. For a fixed-effects ANOVA model with 16 groups, α = 0.05, and 95% power, at least six specimens per group were required. The sample size was therefore set at eight specimens per group.

The materials used in this study, along with their manufacturers and compositions, are listed in Table 1. For each restorative material, specimens with thicknesses of 1.0 and 1.5 mm were prepared. The thicknesses of 1.0 and 1.5 mm were selected to represent clinically relevant thicknesses of monolithic indirect restorations and to enable a controlled assessment of the effect of a 0.5-mm increase in material thickness on light attenuation and the subsequent polymerization of the underlying resin cement. The specimens of VITA Suprinity (VS), VITA Enamic (VE), and Lava Ultimate (LU) were obtained by sectioning the prefabricated blocks using a slow-speed precision cutting machine (Isomet 1000; Buehler, Lake Bluff, IL, USA). After sectioning, the VS specimens were crystallized in accordance with the manufacturer’s instructions (Programat P300, Ivoclar Vivadent AG, Liechtenstein, Austria). Saremco Print Crowntec (SC) specimens were designed with dimensions of (Ø12 mm) and thicknesses of 1.0 or 1.5 mm using CAD software (Autodesk Fusion, v2024; Autodesk Inc., San Francisco, CA, USA)(NS). The Standard Tessellation Language (STL) files were imported into nesting software (exocad 3.2 Elefsina; exocad GmbH, Darmstadt, Germany), and the specimens were fabricated using a DLP-based 3D printer (Sega 3D Printer; 3BFAB Technology Inc., Istanbul, Turkiye). The specimens were printed at a 90° build orientation with a layer thickness of 50 μm. After printing, the specimens were removed from the build platform, and residual resin was removed by washing the specimens in 99% isopropyl alcohol for 5 min using a cleaning unit (Twin Tornado; Medifive, Incheon, Republic of Korea). The specimens were subsequently dried using an air syringe. Post-polymerization was then performed under 385-nm ultraviolet light for 10 min using a curing unit (Twin Cure; Medifive, Incheon, Republic of Korea).

Both surfaces of all restorative material specimens were sequentially wet-polished using 800-, 1000-, and 1200-grit silicon carbide (SiC) abrasive papers to standardize their surface roughness. The thickness of each specimen was verified using a digital caliper (Mitutoyo QSM936; Mitutoyo America Corporation, Aurora, IL, USA) to confirm the final thicknesses of 1.0 and 1.5 mm. All specimens were ultrasonically cleaned in distilled water for 10 min using an ultrasonic cleaner (EasyClean Ultrasonic Cleaner; Renfert GmbH & Co., Hilzingen, Germany) and air-dried.

### 2.1. Resin Cement Specimen Preparation

**Figure 1:**
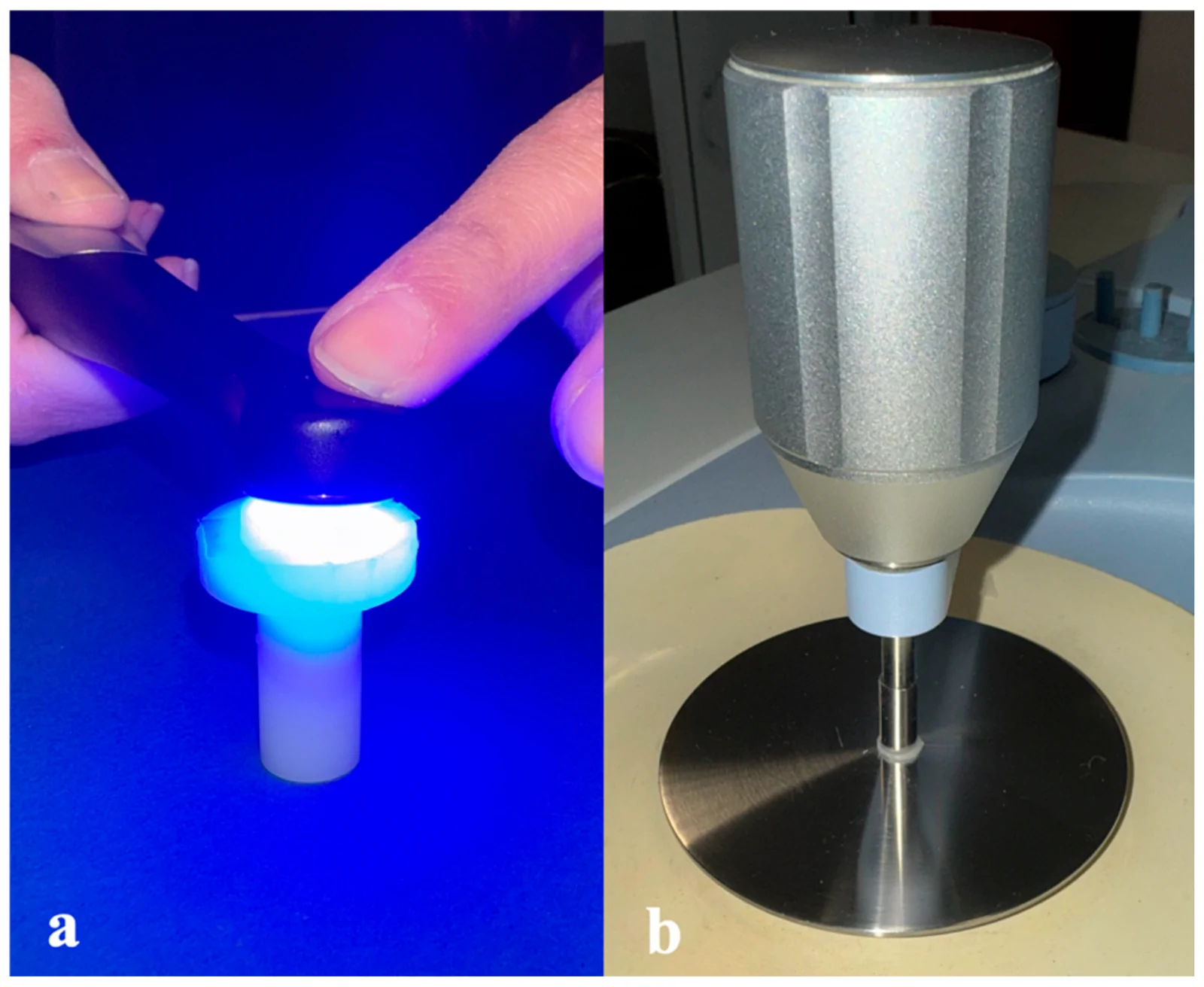
Polymerization of the resin cement specimen using the light-curing unit (**a**), measurement of the degree of conversion of the resin cement specimen using FT-IR/ATR (**b**).

Resin cement specimens were prepared using a custom-made polytetrafluoroethylene (Teflon) mold with an internal diameter of 8 mm and a height of 0.8 mm. The mold was positioned on a glass slide to provide a flat surface. A dual-cure self-adhesive resin cement (Nova Resin Universal shade, Imicryl Dental, Konya, Turkiye) was injected into the mold and covered with a transparent polyester strip to minimize oxygen inhibition. The restorative material specimen assigned to the experimental group was then positioned over the polyester strip. Polymerization was performed through the restorative material using the VALO X curing unit in SP or XP mode (Figure 1a). During light curing, the curing-light tip was positioned perpendicular to and in contact with the restorative material surface. The resin cement specimens were removed from the mold and stored under dry and dark conditions at 23 ± 2 °C until DC analysis. A total of 128 resin cement specimens were prepared according to restorative material type, material thickness (1.0 and 1.5 mm), and curing mode (SP and XP), with 8 specimens included in each experimental group.

### 2.2. Transmitted Irradiance Measurement

The curing unit (VALO X; Ultradent Products Inc., South Jordan, UT, USA), equipped with a 12 mm-diameter lens aperture, was operated in Standard Power (SP) mode for 10 s and Xtra Power (XP) mode for 5 s. These exposure times were selected in accordance with the manufacturer’s recommended protocol for each curing mode. According to the manufacturer, SP provides continuous irradiation, whereas XP delivers a triple-pulse exposure with a 0.5-s automatic rest interval between pulses. Baseline irradiance was initially measured in both curing modes without an intervening restorative material. For the transmitted irradiance measurements, each restorative material specimen was placed directly on the sensor, and the curing-light tip was positioned perpendicular to and in contact with the upper surface of the specimen. Three consecutive technical measurements were obtained using the same specimen for each material–thickness–curing mode combination. Measurements displayed in W/cm^2^ were converted to mW/cm^2^, and the results were expressed as mean ± standard deviation. Calculated radiant exposure (J/cm^2^) was determined by multiplying the mean transmitted irradiance (mW/cm^2^) by the manufacturer-specified exposure time for the corresponding mode (10 s for SP and 5 s for XP) and dividing the result by 1000.

$$Radiant\;exposure(J/{cm}^{2})=((Mean\;transmitted\;irradiance(mW/{cm}^{2}))\times (Exposure\;time(s)))/1000$$

Because the XP irradiance values represented the mean irradiance over the complete pulsed curing cycle, the 0.5-s automatic rest intervals between pulses were already reflected in the measurements and were therefore not subtracted separately from the manufacturer-specified total exposure time.

### 2.3. Translucency Parameter Measurement

Color measurements were performed under D65 illumination using a spectrophotometer with an integrated light source and a 5 mm measuring tip (VITA Easyshade V; VITA Zahnfabrik, Bad Säckingen, Germany). A single operator (SK) measured each specimen over white (L* = 96.7, a* = 3.5, b* = 8.5) and black (L* = 2.9, a* = 0.8, b* = 2.1) backgrounds. Each measurement was repeated three times over each background, and the mean L*, a*, and b* values were used for the calculation of RTP_00_ [19,20,35]. The RTP_00_ was calculated by applying the CIEDE2000 color-difference formula to the color coordinates obtained over the black and white backgrounds, with the subscripts B and W referring to the black and white backgrounds, respectively. The calculated RTP_00_ values were used to descriptively characterize the translucency of the restorative materials at each thickness [20,36].

$$RTP_{00}={\left[{\left(\frac{L_{B}^{\prime }-L_{W}^{\prime }}{K_{L}S_{L}}\right)}^{2}+{\left(\frac{C_{B}^{\prime }-C_{W}^{\prime }}{K_{C}S_{C}}\right)}^{2}+{\left(\frac{H_{B}^{\prime }-H_{W}^{\prime }}{K_{H}S_{H}}\right)}^{2}+R_{T}\left(\frac{C_{B}^{\prime }-C_{W}^{\prime }}{K_{C}S_{C}}\right)\left(\frac{H_{B}^{\prime }-H_{W}^{\prime }}{K_{H}S_{H}}\right)\right]}^{1/2}$$

### 2.4. Degree of Conversion Analysis

**Figure 2:**
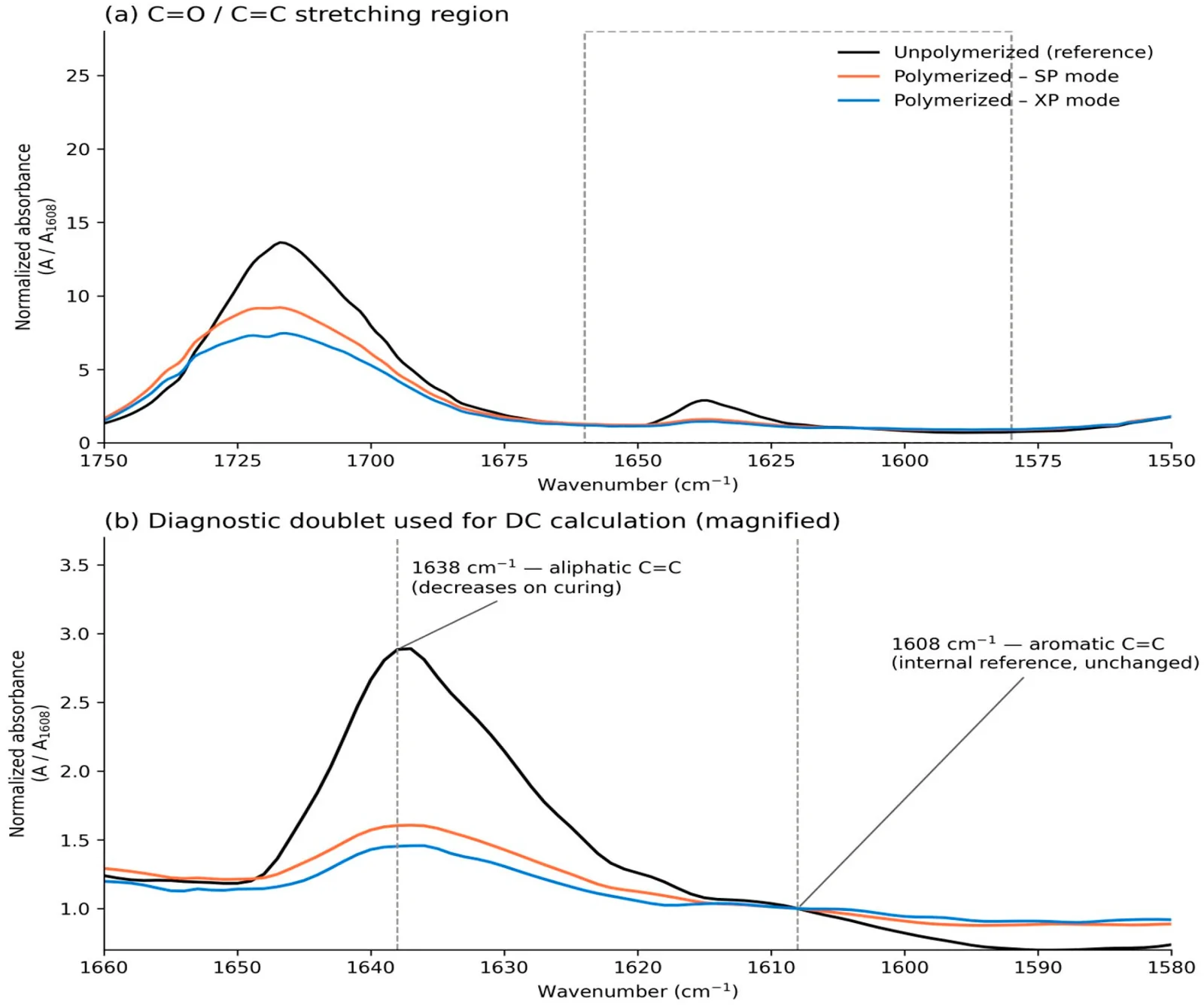
Representative FT-IR/ATR spectra of the dual-cure resin cement before polymerization and after polymerization using Standard Power (SP) and Xtra Power (XP) curing modes. (**a**) Spectra in the C=O/C=C stretching region (1750–1550 cm^−1^). (**b**) Magnified view of the diagnostic region showing the aliphatic C=C peak at 1638 cm^−1^ and the aromatic C=C internal reference peak at 1608 cm^−1^ used for degree of conversion calculations. The reduction in the 1638 cm^−1^ peak relative to the 1608 cm^−1^ reference peak indicates methacrylate double-bond conversion.

The DC of the resin cement was evaluated using FT-IR/ATR spectroscopy (Spectrum 100; PerkinElmer, Waltham, MA, USA) at 1 and 10 days after polymerization (Figure 1b). A background spectrum was recorded before the measurements. An uncured resin cement spectrum was obtained and used as the unpolymerized reference. One spectrum was recorded from the center of the bottom surface of each polymerized resin cement specimen, which faced away from the curing light. Spectra were collected over a range of 4000–650 cm^−1^ at a resolution of 4 cm^−1^, using four scans per spectrum. Prior to peak-height measurements, all spectra were baseline-corrected using the automatic baseline-correction function of Spectrum software (v1.60; PerkinElmer, Waltham, MA, USA). The corrected spectra were subsequently inspected, and residual baseline distortions in the diagnostic region containing the peaks at 1638 and 1608 cm^−1^ were manually corrected when necessary. Peak heights were then measured relative to the corrected baseline using the same procedure for all spectra. DC was determined using the two-frequency method based on the absorbance peak-height ratio of the aliphatic C=C bond at 1638 cm^−1^ to the aromatic C=C reference peak at 1608 cm^−1^ (Figure 2). DC was calculated using the following equation:

DC (%) = [1 − (A_1638_/A_1608_) polymerized/(A_1638_/A_1608_) uncured] × 100

where A_1638_ and A_1608_ represent the absorbance peak heights at 1638 and 1608 cm^−1^, respectively. The same specimens were remeasured on day 10 using the same protocol. Between the day-1 and day-10 measurements, the specimens were stored under dry and dark conditions at 23 ± 2 °C. Specimen preparation and FT-IR/ATR measurements were performed in a randomized order; however, the operator was not blinded to group allocation [37].

### 2.5. Statistical Analysis

The normality of the data was assessed using the Shapiro–Wilk test, and the homogeneity of variances was evaluated using Levene’s test. The effects of restorative material type, material thickness, curing mode, and post-irradiation time on DC were analyzed using a mixed-design repeated-measures ANOVA. Restorative material type, material thickness, and curing mode were included as between-subject factors, whereas post-irradiation time was included as the within-subject factor. When significant differences were detected, pairwise comparisons were performed using Tukey’s HSD post hoc test. The results are presented as mean ± standard deviation, with letter-based groupings generated separately for the 1- and 10-day measurements. All analyses were performed using IBM SPSS Statistics for Windows, version 22.0 (IBM Corp., Armonk, NY, USA), and the significance level was set at α = 0.05.

## 3. Results

The Shapiro–Wilk and Levene tests indicated that the assumptions of normality and homogeneity of variances were satisfied (*p* > 0.05). The mixed-design repeated-measures ANOVA revealed significant main effects of restorative material type (*p* < 0.001) and material thickness (*p* < 0.001) on DC. Curing mode had no significant effect on DC (*p* = 0.110), whereas post-irradiation time had a significant effect (*p* < 0.001). A significant interaction was observed between restorative material type and material thickness (*p* = 0.043). None of the remaining interactions were statistically significant (*p* > 0.05) (Table 2).

**Figure 3:**
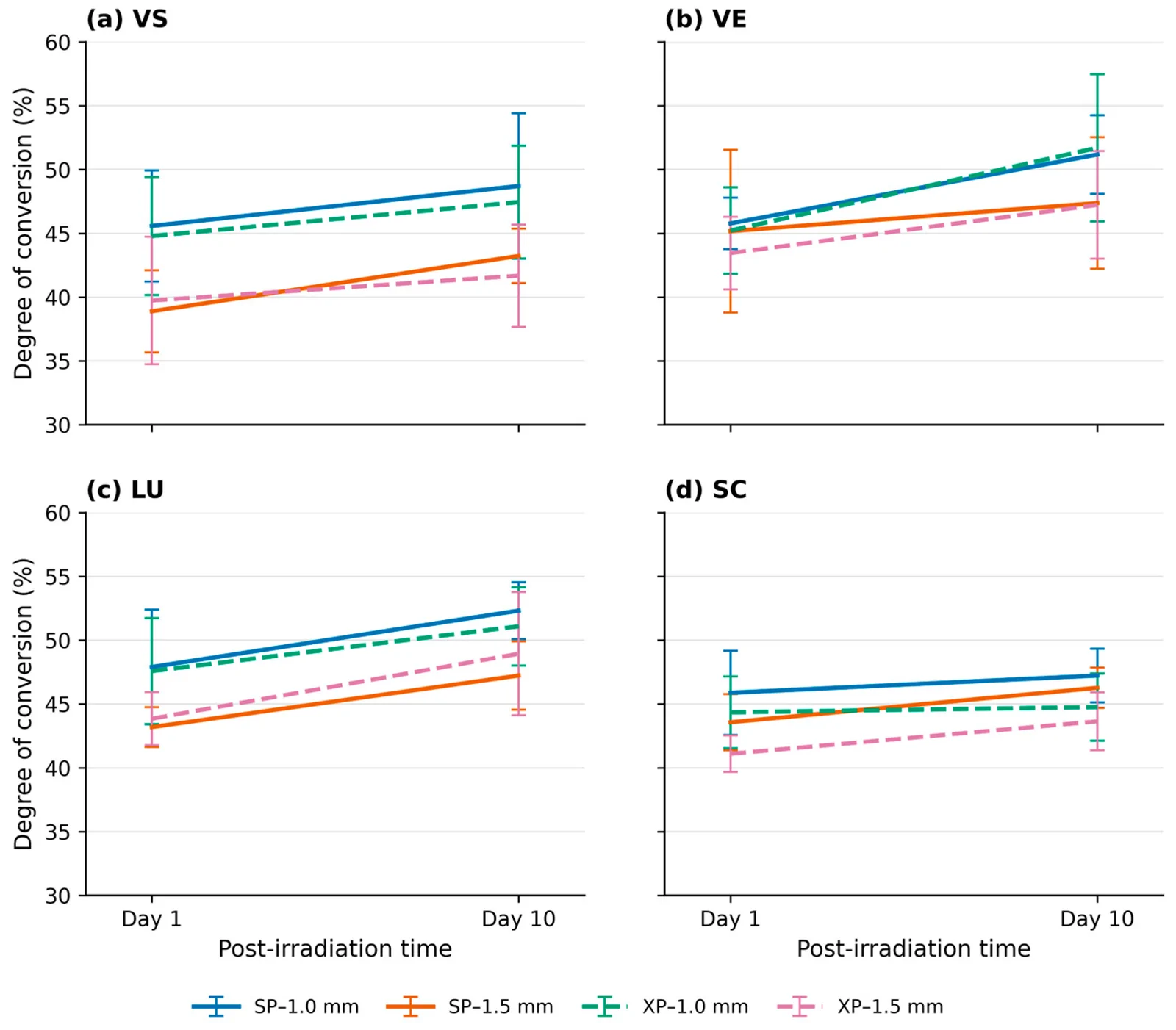
Changes in mean degree of conversion (DC) according to restorative material, material thickness, curing mode, and post-irradiation time. (**a**) VITA Suprinity (VS); (**b**) VITA Enamic (VE); (**c**) Lava Ultimate (LU); and (**d**) Saremco Print Crowntec (SC). Error bars indicate standard deviations.

The mean DC values according to restorative material type, material thickness, curing mode, and post-irradiation time are presented in Table 3. DC increased from day 1 to day 10 in all material-thickness-curing mode combinations. On day 1, the numerically highest mean DC was observed for LU at 1.0 mm under SP mode (47.90 ± 4.49%), whereas the numerically lowest mean was observed for VS at 1.5 mm under SP mode (38.89 ± 3.22%). On day 10, the numerically highest mean DC was again observed for LU at 1.0 mm under SP mode (52.32 ± 2.23%), whereas the numerically lowest mean was observed for VS at 1.5 mm under XP mode (41.68 ± 4.01%) (Table 3). In the VS group, the difference between thickness groups polymerized with the SP mode was statistically significant at day 1 (*p* < 0.05), but not at day 10. No other within-mode thickness comparisons reached statistical significance for VS, VE, LU, or SC at either time point (Table 3). The changes in DC according to restorative material type, thickness, curing mode, and post-irradiation time are illustrated in Figure 3.

The mean baseline irradiance was 1402 ± 9 mW/cm^2^ in SP mode and 2734 ± 60 mW/cm^2^ in XP mode. At 1.0 mm, LU exhibited the highest transmitted irradiance and VS the lowest in both curing modes. At 1.5 mm, the highest values were observed for VE, whereas VS again exhibited the lowest values. An increase in material thickness reduced the transmitted light intensity for all materials in both curing modes; however, with the exception of VE in XP mode, the values in this mode were found to be similar (Table 4). Although XP generally produced higher transmitted irradiance values than SP, its shorter manufacturer-specified exposure time resulted in lower calculated radiant exposure values after transmission through the restorative materials (Table 4).

At a thickness of 1.0 mm, LU exhibited the highest mean RTP_00_ value (20.06 ± 0.09), whereas VS exhibited the lowest value (16.79 ± 0.08). At 1.5 mm, the highest mean RTP_00_ value was observed for VS (15.59 ± 2.89), while SC exhibited the lowest value (9.28 ± 1.78). RTP_00_ decreased with increasing thickness in all restorative materials, with the greatest numerical reduction observed for SC (Table 5).

## 4. Discussion

The findings of this study demonstrated that restorative material type and material thickness significantly influenced the DC of the dual-cure resin cement, with a significant interaction between these two factors. Curing mode had no significant effect on DC, whereas post-irradiation time significantly affected DC, with values increasing from day 1 to day 10. Accordingly, the null hypothesis was partially rejected.

The four tested materials differ considerably in composition: VS is a zirconium oxide-reinforced lithium silicate ceramic [38]; VE is a hybrid ceramic composed of a feldspathic ceramic network infiltrated with a methacrylate polymer [39]; LU is a resin nanoceramic containing silica and zirconium oxide nanoparticles within a resin matrix [38]; and SC is a 3D-printed resin composite containing an inorganic filler within a dimethacrylate resin matrix [40]. LU exhibited the highest DC values, particularly at 1 mm, whereas VS exhibited the lowest DC values, particularly at 1.5 mm. VE and SC generally showed intermediate values, although VE showed values close to LU at 1.5 mm. Differences in the proportion and composition of the organic (resin) and inorganic (ceramic/filler) phases, together with the potential lack of a homogeneous structure in specimens fabricated from the 3D-printed material, may account for the observed differences in DC among the materials. Similarly, Boztuna Çetindemir et al., who compared different restorative material types (zirconia-reinforced lithium silicate, hybrid ceramic, nanoceramic, and monolithic zirconia), reported that the DC of the resin cement varied significantly according to the amount, size, and type of crystalline structure of the material [23].

Beyond composition, material thickness is another key determinant of light transmittance. In the present study, an increase in material thickness was found to reduce the DC. As thickness increases, light absorption and scattering within the material also increase, thereby reducing the amount of light reaching the resin cement [17]. Consequently, the activation of photoinitiator systems is limited, resulting in lower DC. Egilmez et al., in a study evaluating the effect of different CAD/CAM materials and material thicknesses on the DC of dual-cure resin cement, reported that material type influenced light transmittance, but that the DC was primarily affected by material thickness. Furthermore, it has been reported in the literature that increasing thickness alters the optical behavior of the material by reducing both translucency and transmitted irradiance [17,27,32]. Cengiz et al., in a study conducted with CAD/CAM hybrid ceramics of different thicknesses, demonstrated that translucency decreased as substrate thickness increased, and that even small changes in thickness could cause clinically perceptible optical differences [3]. Similarly, Pop-Ciutrila et al. reported that increases in ceramic thickness above 0.9 mm reduced the translucency parameter, leading to noticeable optical differences [41]. This reduction in translucency also directly affects the amount of irradiance reaching the underlying resin cement; indeed, Borges et al. reported that the thickness, shade, and translucency of lithium disilicate ceramics significantly affected the transmitted irradiance, with irradiance values decreasing substantially as thickness increased [42]. Consistent with these findings, both translucency and transmitted irradiance decreased with increasing thickness in the present study, and this reduction was accompanied by a corresponding decline in DC. For VS, as material thickness increased, transmitted irradiance decreased markedly, whereas the corresponding reduction in RTP_00_ was limited compared with the other materials. In contrast, for SC, increasing thickness led to a pronounced reduction in both transmitted irradiance and RTP_00_. The decline in translucency and transmitted irradiance with thickness varies depending on the composition, microstructure, filler particle size and distribution, and refractive index match between the organic and inorganic phases of the material.

Curing mode did not significantly affect DC under the tested conditions. Although XP generally produced higher transmitted irradiance than SP, its shorter manufacturer-specified exposure time resulted in lower calculated radiant exposure after transmission through the restorative materials. The calculated baseline radiant exposure values were comparable between XP and SP (13.67 and 14.02 J/cm^2^, respectively). Nevertheless, the observed differences in transmitted irradiance and calculated radiant exposure did not correspond to a statistically significant difference in DC. Consistent with these findings, a previous study using the same curing unit reported that short, high-irradiance XP exposure achieved a depth of cure comparable to that obtained with longer SP exposure in several bulk-fill resin composites [43].

Ayres et al. reported that, when a dual-cure resin cement was polymerized beneath indirect restorative materials of varying thicknesses, the DC measured after 10 min was significantly higher than that measured after 5 min [29]. Kelch et al. found that the DC values of various dual-cure resin composite luting materials increased significantly from baseline to day 2, with no further significant increase observed by day 7 [5]. Carek et al. also demonstrated that the DC of dual-cure resin cements was significantly influenced by the post-irradiation period [12]. Similarly, continued chemical polymerization after light activation may explain the increase in the DC of the dual-cure resin cement from day 1 to day 10 observed in the present study.

This study has certain limitations. It was conducted under in vitro conditions, and temperature fluctuations, moisture, mechanical loading, and aging processes occurring in the oral environment were not simulated. The DC was evaluated only on days 1 and 10, and changes occurring over longer periods were not examined. Furthermore, only a single dual-cure resin cement, a single light-curing unit, and a limited range of material thicknesses (1.0 and 1.5 mm) were evaluated, which may limit the generalizability of the findings to other materials, devices, and clinical conditions. Self-cure-only and restoration-free control groups were not included because the experimental design focused on comparing the effects of restorative material type, thickness, and light-curing mode under indirect-restoration conditions. Nevertheless, the absence of these control groups limited the ability to separately assess the contribution of chemical polymerization and to establish a reference value for the maximum DC achievable under direct light exposure. Further studies incorporating different resin cements, light-curing units, material thicknesses, control groups, and longer evaluation periods are therefore needed.

## 5. Conclusions

Within the limitations of this in vitro study, restorative material type and thickness significantly influenced the DC of the dual-cure resin cement, whereas curing mode (SP vs. XP) had no significant effect at either evaluation time point. The DC increased significantly from day 1 to day 10 across all groups, indicating that post-cure polymerization continued beyond the initial light activation. Increasing material thickness was consistently associated with lower degrees of conversion, underscoring the importance of material selection and thickness control when planning indirect restorations cemented with dual-cure resin cements. Under the specific in vitro conditions tested, SP and XP resulted in comparable DC values; however, these findings should not be generalized to other resin cements, light-curing units, restorative materials, or material thicknesses.

## Figures and Tables

**Table 1:** Restorative materials used in the study, their manufacturers, compositions, material types, and shades.

Material	Manufacturer	Composition	Material Type	Shade
Vita Suprinity (VS) (Zirconia-reinforced lithium silicate ceramic)	VITA Zahnfabrik, Bad Sackingen, Germany	Silicon dioxide, lithium oxide, zirconium dioxide, and phosphorus pentoxide	CAD/CAM milling	A2
Vita Enamic (VE) (Hybrid ceramic)	VITA Zahnfabrik, Bad Sackingen, Germany	14 wt% methacrylate polymer (UDMA, TEGDMA) and 86 wt% fine-structure feldspathic ceramic networks	CAD/CAM milling	A1
Lava Ultimate (LU) (Resin nanoceramic)	3M ESPE, Seefeld, Germany	Bis-GMA, UDMA, Bis-EMA, TEGDMA with 80 wt% silica and zirconia nanoparticles and zirconia/silica nanocluster	CAD/CAM milling	A2
Saremco Print Crowntec (SC) (3D-printed hybrid resin composite)	Saremco Dental AG, Rebstein, Switzerland	Esterification products 4,4′-isopropylidiphenol, ethoxylated and 2-methylprop-2-enoic acid, silanized dental glass, pyrogenic silica, initiators. Total content of inorganic fillers (particle size 0.7 μm) is 30–50% by mass	3D printing	A1
Nova Resin (Dual-curing self-adhesive resin cement)	Imicryl Dental, Konya, Türkiye	Dimethacrylate monomers (Bis-GMA, UDMA, TEGDMA), barium glass/silica fillers, photoinitiator system (camphorquinone-amine), chemical initiator system, pigments and stabilizers	–	Universal

**Table 2:** Results of the Mixed-Design Repeated-Measures ANOVA.

Source	df	F	*p*	Partial η^2^
**Between-subjects effects**				
**Material**	3	14.924	<0.001	0.286
**Thickness**	1	50.452	<0.001	0.311
Mode	1	2.598	0.110	0.023
**Material × Thickness**	3	2.799	0.043	0.070
Material × Mode	3	1.098	0.353	0.029
Thickness × Mode	1	0.085	0.772	0.001
Material × Thickness × Mode	3	0.422	0.738	0.011
Error (Subject/Material × Thickness × Mode)	112	-	-	(MS = 16.089)
**Within-subject effects**				
**Time**	1	66.244	<0.001	0.372
Material × Time	3	2.301	0.081	0.058
Thickness × Time	1	0.012	0.912	<0.001
Mode × Time	1	0.031	0.860	<0.001
Material × Thickness × Time	3	1.472	0.226	0.038
Material × Mode × Time	3	0.488	0.691	0.013
Thickness × Mode × Time	1	0.039	0.844	<0.001
Material × Thickness × Mode × Time	3	0.240	0.868	0.006
Error (Time × Subject/Material × Thickness × Mode)	112	-	-	(MS = 11.014)

Restorative material type, thickness, and curing mode were between-subject factors; post-irradiation time was the within-subject factor. Significant effects are shown in bold (*p* < 0.05).

**Table 3:** Mean DC (%) values by experimental group.

		DC (%) (Day 1)		DC (%) (Day 10)	
		1 mm	1.5 mm	1 mm	1.5 mm
**VS**	SP	45.58 ± 4.36 ^ab^	38.89 ± 3.22 ^c^	48.71 ± 5.70 ^abc^	43.23 ± 2.13 ^cd^
	XP	44.79 ± 4.62 ^abc^	39.73 ± 5.00 ^bc^	47.45 ± 4.41 ^abcd^	41.68 ± 4.01 ^d^
**VE**	SP	45.78 ± 2.02 ^ab^	45.17 ± 6.38 ^abc^	51.18 ± 3.08 ^ab^	47.38 ± 5.15 ^abcd^
	XP	45.22 ± 3.38 ^abc^	43.45 ± 2.85 ^abc^	51.71 ± 5.77 ^a^	47.23 ± 4.22 ^abcd^
**LU**	SP	47.90 ± 4.49 ^a^	43.19 ± 1.56 ^abc^	52.32 ± 2.23 ^a^	47.23 ± 2.68 ^abcd^
	XP	47.58 ± 4.15 ^a^	43.85 ± 2.09 ^abc^	51.09 ± 3.07 ^ab^	48.94 ± 4.83 ^abc^
**SC**	SP	45.88 ± 3.30 ^ab^	43.57 ± 2.20 ^abc^	47.23 ± 2.11 ^abcd^	46.27 ± 1.58 ^abcd^
	XP	44.35 ± 2.82 ^abc^	41.10 ± 1.43 ^bc^	44.76 ± 2.63 ^bcd^	43.64 ± 2.27 ^cd^

Different superscript letters indicate significant differences within the same post-irradiation time (Tukey HSD, *p* < 0.05).

**Table 4:** Mean transmitted irradiance and calculated radiant exposure values by experimental group.

Material	Thickness (mm)	SP İrradiance (mW/cm^2^)	SP Calculated Radiant Exposure (J/cm^2^)	XP İrradiance (mW/cm^2^)	XP Calculated Radiant Exposure (J/cm^2^)
**No material (baseline)**	—	1402 ± 9	14.02 ± 0.09	2734 ± 60	13.67 ± 0.30
**VS**	1.0	752 ± 88	7.52 ± 0.88	961 ± 38	4.81 ± 0.19
**VS**	1.5	423 ± 20	4.23 ± 0.20	512 ± 9	2.56 ± 0.05
**VE**	1.0	869 ± 20	8.69 ± 0.20	1238 ± 106	6.19 ± 0.53
**VE**	1.5	798 ± 37	7.98 ± 0.37	1235 ± 37	6.18 ± 0.19
**LU**	1.0	900 ± 13	9.00 ± 0.13	1344 ± 31	6.72 ± 0.16
**LU**	1.5	780 ± 9	7.80 ± 0.09	1170 ± 69	5.85 ± 0.35
**SC**	1.0	840 ± 18	8.40 ± 0.18	1072 ± 30	5.36 ± 0.15
**SC**	1.5	642 ± 29	6.42 ± 0.29	1006 ± 92	5.03 ± 0.46

Radiant exposure was calculated by multiplying the measured mean transmitted irradiance by the manufacturer-specified exposure time for the corresponding curing mode (10 s for SP and 5 s for XP) and dividing the result by 1000.

**Table 5:** Mean RTP_00_ values by restorative material and thickness.

	Thickness	
Material	1.0 mm	1.5 mm
**VS**	16.79 ± 0.08	15.59 ± 2.89
**VE**	17.34 ± 0.39	13.06 ± 0.71
**LU**	20.06 ± 0.09	15.27 ± 0.08
**SC**	18.40 ± 0.73	9.28 ± 1.78

## Data Availability

The original contributions presented in this study are included in the article. Further inquiries can be directed to the corresponding author.

## Abbreviations

The following abbreviations are used in this manuscript:

CAD/CAM	Computer-Aided Design/Computer-Aided Manufacturing
DC	Degree of Conversion
SP	Standard Power
XP	Xtra Power
VS	VITA Suprinity
VE	VITA Enamic
LU	Lava Ultimate
SC	Saremco Print Crowntec
RTP_00_	Relative Translucency Parameter
CIEDE2000	CIE 2000 Color Difference Formula
FT-IR/ATR	Fourier-Transform Infrared/Attenuated Total Reflectance Spectroscopy
ANOVA	Analysis of Variance
HSD	Honestly Significant Difference

## References

[1] Cioloca Holban C., Tatarciuc M., Vitalariu A.M., Vasluianu R.-I., Antohe M., Diaconu D.A., Stamatin O., Dima A.M. (2025). Three-dimensional printing and CAD/CAM milling in prosthodontics: A scoping review of key metrics towards future perspectives. J. Clin. Med..

[2] Németh A., Teutsch B., Szabó B., Borbély J. (2024). Comparison of Additive and Subtractive Manufacturing for Fixed Dental Restorations: A Systematic Review and Meta-Analysis. J. Dent..

[3] Yalcinkaya Cengiz H., Çölgeçen Ö., Uzer Celik E. (2026). Evaluation of the degree of conversion of luting materials beneath hybrid ceramic CAD/CAM substrates of different thicknesses. BMC Oral Health.

[4] De Souza G., Braga R.R., Cesar P.F., Lopes G.C. (2015). Correlation between clinical performance and degree of conversion of resin cements: A literature review. J. Appl. Oral Sci..

[5] Kelch M., Stawarczyk B., Mayinger F. (2022). Time-dependent degree of conversion, Martens parameters, and flexural strength of different dual-polymerizing resin composite luting materials. Clin. Oral Investig..

[6] Xu S., Chen L., Lin X., Yang X., He L., Yan S., Luo S., Chen X., Que G. (2024). Resin monomers induce apoptosis of the pulp–dentin complex through the mitochondrial pathway. J. Toxicol. Sci..

[7] Chang H.-H., Chang M.-C., Wang H.-H., Huang G.-F., Lee Y.-L., Wang Y.-L., Chan C.-P., Yeung S.-Y., Tseng S.-K., Jeng J.-H. (2014). Urethane dimethacrylate induces cytotoxicity and regulates cyclooxygenase-2, hemeoxygenase and carboxylesterase expression in human dental pulp cells. Acta Biomater..

[8] Chang C.-Y., Chiang C.-Y., Chiang Y.-W., Lee M.-W., Lee C.-Y., Chen H.-Y., Lin H.-W., Kuan Y.-H. (2020). Toxic effects of urethane dimethacrylate on macrophages through caspase activation, mitochondrial dysfunction, and reactive oxygen species generation. Polymers.

[9] Almeida J.R., Schmitt G.U., Kaizer M.R., Boscato N., Moraes R.R. (2015). Resin-based luting agents and color stability of bonded ceramic veneers. J. Prosthet. Dent..

[10] Martins F.V., Vasques W.F., Fonseca E.M. (2019). How the variations of the thickness in ceramic restorations of lithium disilicate and the use of different photopolymerizers influence the degree of conversion of the resin cements: A systematic review and meta-analysis. J. Prosthodont..

[11] Oh S., Shin S.-M., Kim H.-J., Paek J., Kim S.-J., Yoon T.H., Kim S.-Y. (2018). Influence of glass-based dental ceramic type and thickness with identical shade on the light transmittance and the degree of conversion of resin cement. Int. J. Oral Sci..

[12] Carek A., Dukaric K., Miler H., Marovic D., Tarle Z., Par M. (2022). Post-cure development of the degree of conversion and mechanical properties of dual-curing resin cements. Polymers.

[13] Ikemoto S., Komagata Y., Yoshii S., Masaki C., Hosokawa R., Ikeda H. (2024). Impact of CAD/CAM material thickness and translucency on the polymerization of dual-cure resin cement in endocrowns. Polymers.

[14] Es Sebar L., Baldi A., Comba A., Sannino I., Iannucci L., Grassini S., Shokuhfar T., Scotti N. (2025). Could tack-curing influence margin continuity and conversion degree of a universal dual-curing cement?. Materials.

[15] Li Q., Lin H.-L., Zheng M., Ozcan M., Yu H. (2021). Minimum radiant exposure and irradiance for triggering adequate polymerization of a photo-polymerized resin cement. Materials.

[16] de Mendonça L.M., Ramalho I.S., Lima L.A.S.N., Pires L.A., Pegoraro T.A., Pegoraro L.F. (2019). Influence of the composition and shades of ceramics on light transmission and degree of conversion of dual-cured resin cements. J. Appl. Oral Sci..

[17] Wu Z., Tian J., Wei D., Zhang Y., Lin Y., Di P. (2023). Effects of thickness and polishing treatment on the translucency and opalescence of six dental CAD-CAM monolithic restorative materials: An in vitro study. BMC Oral Health.

[18] Lee Y.-K. (2007). Influence of scattering/absorption characteristics on the color of resin composites. Dent. Mater..

[19] Johnston W.M., Reisbick M.H. (1997). Color and translucency changes during and after curing of esthetic restorative materials. Dent. Mater..

[20] Kürklü D., Azer S.S., Yilmaz B., Johnston W.M. (2013). Porcelain thickness and cement shade effects on the colour and translucency of porcelain veneering materials. J. Dent..

[21] Al-Zain A.O., Eckert G.J., Platt J.A. (2019). The influence of distance on radiant exposure and degree of conversion using different light-emitting-diode curing units. Oper. Dent..

[22] Sinhoreti M.A.C., Manetta I.P., Tango R.N., Iriyama N.T., Consani R.L.X., Correr-Sobrinho L. (2007). Effect of light-curing methods on resin cement Knoop hardness at different depths. Braz. Dent. J..

[23] Çetindemir A.B., Şermet B., Öngül D. (2018). The effect of light sources and CAD/CAM monolithic blocks on degree of conversion of cement. J. Adv. Prosthodont..

[24] Ilday N.O., Bayindir Y.Z., Bayindir F., Gurpinar A. (2013). The effect of light curing units, curing time, and veneering materials on resin cement microhardness. J. Dent. Sci..

[25] Korkut B., Saygili C.C., Murat N., Bayraktar E.T., Beolchi R.S., Tarcin B. (2025). Comparison of dental curing units and output modes regarding radiant flux, tip diameter, radiant emittance, scattering, and penetration depth. Clin. Oral Investig..

[26] Chen T.-A., Lu P.-Y., Lin P.-Y., Chi C.-W., Cheng H.Y., Lai Y.-J., Wang F., Chiang Y.-C. (2023). Effects of ceramic thickness, ceramic translucency, and light transmission on light-cured bulk-fill resin composites as luting cement of lithium disilicate based-ceramics. J. Prosthodont. Res..

[27] Doğu Kaya B., Öztürk S., Şenol A.A., Kahramanoğlu E., Yılmaz Atalı P., Tarçın B. (2024). Effect of CAD-CAM block thickness and translucency on the polymerization of luting materials. BMC Oral Health.

[28] Babaier R., Haider J., Silikas N., Watts D.C. (2022). Effect of CAD/CAM aesthetic material thickness and translucency on the polymerisation of light-and dual-cured resin cements. Dent. Mater..

[29] Ayres A.P.A., Andre C.B., Pacheco R.R., Carvalho A.O., Bacelar-Sá R.C., Rueggeberg F.A., Giannini M. (2015). Indirect restoration thickness and time after light-activation effects on degree of conversion of resin cement. Braz. Dent. J..

[30] Pacheco R.R., Carvalho A.O., André C.B., Ayres A.P.A., de Sá R.B.C., Dias T.M., Rueggeberg F.A., Giannini M. (2019). Effect of indirect restorative material and thickness on light transmission at different wavelengths. J. Prosthodont. Res..

[31] Floriani F., Abuhammoud S., Rojas-Rueda S., Unnadkat A., Fischer N.G., Fu C.-C., Jurado C.A. (2024). The Influence of Thickness on Light Transmission for Pre-and Fully Crystallized Chairside CAD/CAM Lithium Disilicate Ceramics. Materials.

[32] Egilmez F., Ergun G., Cekic-Nagas I., Vallittu P.K., Lassila L.V.J. (2017). Light Transmission of Novel CAD/CAM Materials and Their Influence on the Degree of Conversion of a Dualcuring Resin Cement. J. Adhes. Dent..

[33] Liporoni P.C., Ponce A.C., de Freitas M.R., Zanatta R.F., Pereira M.C., Catelan A. (2020). Influence of thickness and translucency of lithium disilicate ceramic on degree of conversion of resinous materials. J. Clin. Exp. Dent..

[34] Jacobs W., Camargo B., Ahmed M., Willems E., Čokić S.M., Zhang F., Vleugels J., Van Meerbeek B. (2025). Light-curing of restorative composite through milled and 3D-printed full-contour zirconia for adhesive luting. Dent. Mater..

[35] Salas M., Lucena C., Herrera L.J., Yebra A., Della Bona A., Pérez M.M. (2018). Translucency thresholds for dental materials. Dent. Mater..

[36] Luo M.R., Cui G., Rigg B. (2001). The development of the CIE 2000 colour-difference formula: CIEDE2000. Color Res. Appl..

[37] Palta N., Secilmis A., Yazicioglu H. (2016). Effect of monolithic zirconia on the degree of conversion of two resin cements analyzed by FT-IR/ATR spectroscopy. J. Adhes. Sci. Technol..

[38] Belli R., Wendler M., de Ligny D., Cicconi M.R., Petschelt A., Peterlik H., Lohbauer U. (2017). Chairside CAD/CAM materials. Part 1: Measurement of elastic constants and microstructural characterization. Dent. Mater..

[39] El Zhawi H., Kaizer M.R., Chughtai A., Moraes R.R., Zhang Y. (2016). Polymer infiltrated ceramic network structures for resistance to fatigue fracture and wear. Dent. Mater..

[40] Duarte J.S., Phark J. (2025). Advances in Dental Restorations: A Comprehensive Review of Machinable and 3D-Printed Ceramic-Reinforced Composites. J. Esthet. Restor. Dent..

[41] Pop-Ciutrila I.S., Ghinea R., Dudea D., Ruiz-López J., Pérez M.M., Colosi H. (2021). The effects of thickness and shade on translucency parameters of contemporary, esthetic dental ceramics. J. Esthet. Restor. Dent..

[42] Borges L.P.S., Borges G.A., Correr A.B., Platt J.A., Kina S., Correr-Sobrinho L., Costa A.R. (2021). Effect of lithium disilicate ceramic thickness, shade and translucency on transmitted irradiance and knoop microhardness of a light cured luting resin cement. J. Mater. Sci. Mater. Med..

[43] Dos Santos D.B., de Cássia Romano B., Pecorari V.G.A., Price R.B., Giannini M. (2024). Effect of high irradiance and short exposure times on the depth of cure of six bulk-fill resin composites. Eur. J. Oral Sci..

